# Intercellular Transfer of Oncogenic *KRAS* via Tunneling Nanotubes Introduces Intracellular Mutational Heterogeneity in Colon Cancer Cells

**DOI:** 10.3390/cancers11070892

**Published:** 2019-06-26

**Authors:** Snider Desir, Phillip Wong, Thomas Turbyville, De Chen, Mihir Shetty, Christopher Clark, Edward Zhai, Yevgeniy Romin, Katia Manova-Todorova, Timothy K. Starr, Dwight V. Nissley, Clifford J. Steer, Subbaya Subramanian, Emil Lou

**Affiliations:** 1 Department of Medicine, Division of Hematology, Oncology and Transplantation, University of Minnesota, Minneapolis, MN 55455, USA; 2 Department of Integrative Biology and Physiology, University of Minnesota, Minneapolis, MN 55455, USA; 3 Department of Medicine, Division of Gastroenterology, Hepatology and Nutrition, University of Minnesota, Minneapolis, MN 55455, USA; 4 NCI RAS Initiative, Cancer Research Technology Program, Frederick National Laboratory for Cancer Research, Frederick, MD 21702, USA; 5 Department of Obstetrics and Gynecology, Division of Gynecologic Oncology, University of Minnesota, Minneapolis, MN 55455, USA; 6 Molecular Cytology Core Facility, Memorial Sloan-Kettering Cancer Center, New York, NY 10065, USA; 7 Department of Surgery, University of Minnesota, Minneapolis, MN 55455, USA

**Keywords:** tunneling nanotubes, intercellular communication, *KRAS*, oncogene, cellular reprogramming, intercellular transfer, colon cancer, colorectal cancer, confocal microscopy

## Abstract

Mutated forms of the RAS oncogene drive 30% of all cancers, but they cannot be targeted therapeutically using currently available drugs. The molecular and cellular mechanisms that create a heterogenous tumor environment harboring both mutant and wild-type RAS have not been elucidated. In this study, we examined horizontal transfer of mutant *KRAS* between colorectal cancer (CRC) cells via a direct form of cell-to-cell communication called tunneling nanotubes (TNTs). TNT formation was significantly higher in CRC cell lines expressing mutant *KRAS* than CRC cell lines expressing wild-type RAS; this effect was most pronounced in metastatic CRC cell lines with both mutant *KRAS* and deficiency in mismatch repair proteins. Using inverted and confocal fluorescence time-lapse and fluorescence recovery after photobleaching (FRAP)-based microscopy, we observed GFP-tagged mutant *KRAS*^G12D^ protein trafficking between CRC cells through TNTs within a span of seconds to several minutes. Notably, acquisition of mutant *KRAS* increased Extracellular Signal-regulated Kinase (ERK) phosphorylation and upregulated tunneling nanotube formation in recipient wildtype CRC cells. In conclusion, these findings suggest that intercellular horizontal transfer of RAS can occur by TNTs. We propose that intercellular transfer of mutant RAS can potentially induce intratumoral heterogeneity and result in a more invasive phenotype in recipient cells.

## 1. Introduction

RAS is a ubiquitous oncogene in cancers and is highly active and prevalent in both pancreatic (90–95% *KRAS* mutations) and colorectal cancers (CRC) (35–40%). *KRAS* acts as a critical driving force in these cancers, as mutated forms of *KRAS* are constitutively activated, permitting significant downstream effects including increased cell proliferation, tumor progression, and higher rates of metastasis [1,2,3,4,5,6]. There is also increasing evidence that mutated versions of *KRAS* lead to the development of chemoresistance and that subclones of mutated *KRAS* are present at the time of diagnosis of CRC even in tumors that are initially identified as wild-type (wt) for *KRAS* [7].

It has been shown that mutant *KRAS* subclones that arise early in tumorigenesis confer selective growth advantages for tumors as a whole, including drug resistance [8]. Furthermore, the proportion of mutant *KRAS* subclones can vary widely between tumors, and the spatial distribution of these subclones is associated with the most invasive regions of CRC tumors [8]. The current paradigm of emergence of *KRAS*-driven tumors relies on the premise that: (i) mutant *KRAS* arises in the setting of several potential risk factors, including aging and tobacco use; and (ii) cells that acquire mutant *KRAS* do so only in a replicative state from parent cells (i.e., vertical transmission). Horizontal transmission, however, provides an additional means by which cells within a defined tumor can share mutant molecular signals [9,10,11]. RAS itself has been shown to be transferred between cells via exosomes, propagating long-range cellular communication via a diffusible mechanism [12,13,14]. Further, intercellular transfer of the oncogenic H-Ras subclass has been shown to occur between B and T cell lymphocytes, providing additional insight into the role of intercellular communication on antigen-presenting cells in general and also potential implications of transfer of RAS specifically [15,16]. Intratumoral heterogeneity of *KRAS*, in which multiple alleles of the oncogene exist within an individual tumor, can lead to the misdiagnosis of tumors as wild-type RAS. Such tumors, when treated with monoclonal antibody targeting the epidermal growth factor receptor (EGFR), will eventually develop resistance to anti-EGFR therapy, a characteristic most prominent in colorectal cancer [17,18]. We hypothesized that tunneling nanotubes (TNTs) provide an additional mechanism of intercellular communication of oncogenic *KRAS* among colon cancer cells. Intercellular transfer mediated by TNTs presents a new paradigm in which mutant oncogenic proteins, such as RAS, can be directly transmitted horizontally from cell to cell within tumors, thus inducing a greater state of intracellular and also intratumoral heterogeneity.

TNTs are ultrafine, long, filamentous actin-based protrusions of the cell plasma membrane. Characteristic morphologic properties include: (i) their non-adherence to the substratum when observed in in vitro cell culture; (ii) a relatively narrow diameter compared with other actin-based cell protrusions (50–800 nm); and (iii) lengths that can exceed 10-fold the diameter of TNT-forming cells [9,19,20]. TNTs have been shown to mediate intercellular redistribution and sharing of proteins, genetic materials including microRNAs and siRNAs, and other cytoplasmic cargo between cells [10,11,21,22]. We have also previously shown that tumor-derived exosomes can induce cells to upregulate formation of TNTs and utilize them as direct intercellular means for transport [23]. TNTs have been imaged in human and mouse model tumors extensively by our group and others using confocal fluorescence and other forms of high-resolution microscopy [10,11,24]. We recently reported the presence of TNTs connecting cells in tumor tissues obtained from colon cancer patients, in addition to other invasive malignancies [25]. Here we show that TNTs mediate intercellular transfer of mutant *KRAS* in recipient colon cancer cells, thus facilitating intracellular and molecular heterogeneity in the tumor microenvironment. 

## 2. Results

### 2.1. Increased TNT Formation in CRC Cells Harboring Mutant KRAS and Deficient Mismatch Repair

We have previously found that the rate of TNT formation is heterogeneous and variable even among cancer types of similar tissue of origin. For this study, we hypothesized that colon carcinoma cells form TNTs at rates that vary based on *KRAS* status (wild type vs. mutant) and site of origin (i.e., cells derived from a primary CRC tumor vs. metastatic CRC tumors) (Table 1).

We examined TNT formation among five colorectal cancer cell lines and in a colon adenoma cell line (AAC1). The metastatic cell line LOVO and primary colon tumor-derived cell line HCT-116 both endogenously harbor mutant *KRAS*^G13D^; both of these cell lines also are characterized by deficiencies in mismatch repair protein (dMMR), a genetic feature associated with microsatellite instability [30,33,34]. Cell line SW480 harbors mutant *KRAS*^G12V^ variant; and cell lines HCT-8, HT-29, and AAC1 are *KRAS* wild-type (wt) [29,35,36]. HCT-8 also has dMMR. Further details are provided in Table 1.

**Figure 1:**
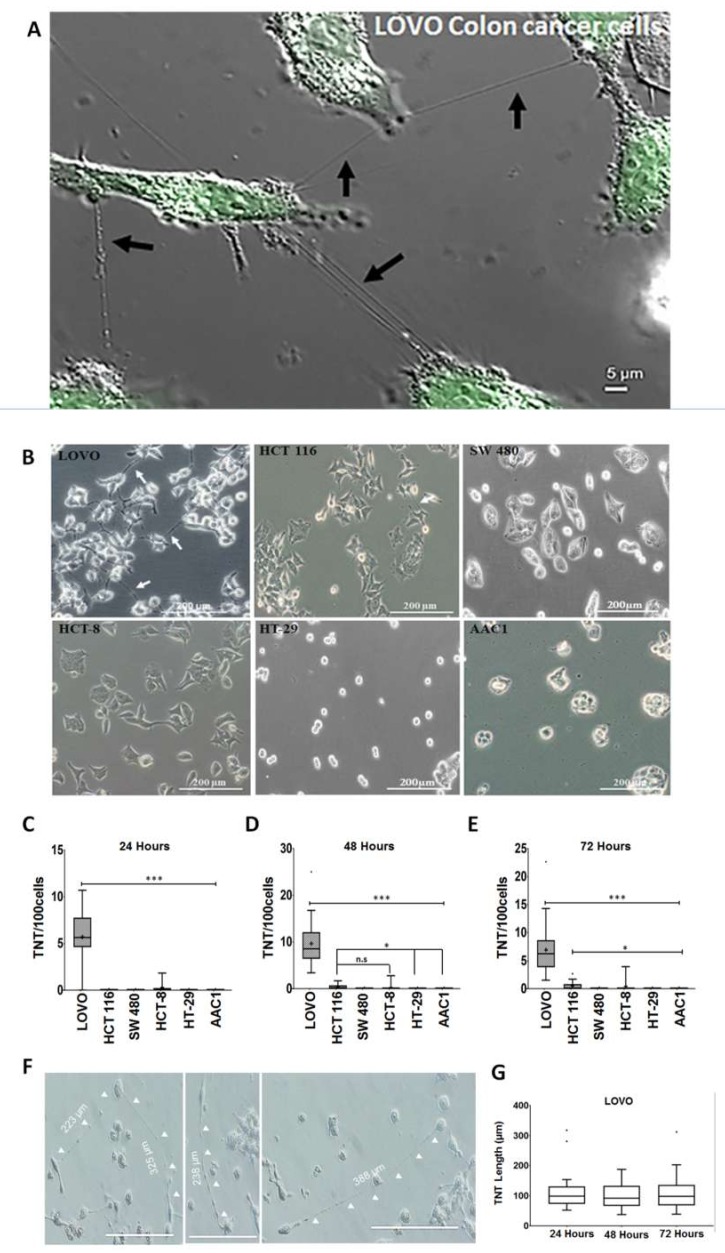
Differential rate of TNT formation among colorectal cancer cells. (**A**) TNTs form to connect colorectal cancer cells in vitro, particularly with invasive cell lines such as LOVO. As shown, TNTs can connect two or more cells to each other, and multiple TNTs can form between the same two cells. Scale bar = 5 µm. (**B**) Representative phase contrast microscopy images of each human colorectal cancer cell line used to quantitatively analyze changes in TNT formation over time (LOVO, HCT 116, SW480, HCT-8, and HT-29 and the premalignant adenoma cell line AAC1). Boxplot representation of TNTs per 100 cells over (**B**) 24 h, (**C**) 48 h, and (**D**) 72 h. Median values for each cell line were compared because of the non-normal distribution of TNT formation. TNTs were manually counted for each cell line over three days using an Olympus IX70 inverted microscope with a 20 × objective lens. 10 fields of views were selected at random in triplicate experiments. (**E**) Phase contrast microscopy images of unusually long TNTs forming between LOVO cells. TNT length was estimated by using Image J to measure image pixels. (**F**) Box plot distribution of LOVO lengths over three days in culture. Symbols on the boxplot are as follows: Box, 1st to 3rd (Q1–Q3) Quartiles; + = Mean; Line inside box = Median. Asterisk symbol indicate statistical differences in median value; n.s = non statistically significant. Scale bar = 200 μm.

We cultured cell lines in sub-confluent conditions for optimal TNT formation (Figure 1A,B) and quantified the number of TNTs and number of cells per high-power field at 24, 48, and 72-hour intervals (Figure 1C–E).

Among the CRC cells tested, LOVO and HCT-116 cells formed the most TNTs (Figure 1). Metastatic LOVO cells formed markedly more TNTs than HCT-116 cells during the entire 72 h period. TNT formation was not evident among SW480, HT-29, or AAC1 cells. Interestingly, LOVO cells did not have the highest proliferation rate (Figure S1A), a characteristic we observed in other cancer lines that form a high number of TNTs [1,2,3]. In addition, the average length of TNTs that formed between LOVO cells did not differ significantly during the 72 h period, with a mean value of 100 µm (Figure 1F–G; additional representative images in Figure S1B). Overall, these findings suggest that there is significant heterogeneity of TNT formation among CRC cell types, with the metastatic LOVO cell line exhibiting the highest rate of TNT formation.

### 2.2. TNTs Facilitate Direct Intercellular Transfer of Oncogenic KRAS between CRC Cells

It is well established that the emergence and expansion of mutant *KRAS* subclones can lead to intratumoral heterogeneity and treatment resistance. Several studies have suggested that mutant *KRAS* can transfer from cell-to-cell [12,15,16,37], but it is unclear whether TNTs have a functional role in the trafficking of mutant *KRAS*.

**Figure 2:**
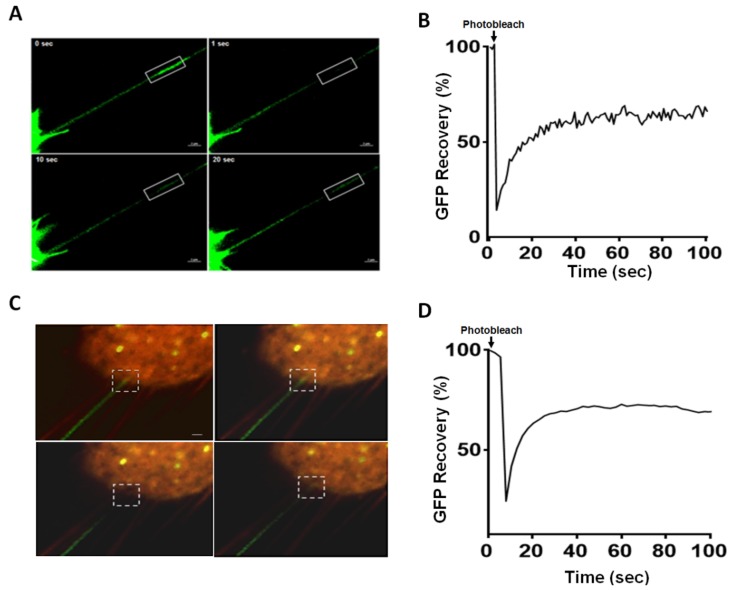
TNTs facilitate intercellular transfer and recipient cell entry of mutant *KRAS*. Photobleaching was performed 24 h after cells were placed in co-culture. Images were captured using a 60 × oil immersion objective lens of a Nikon A1Rsi confocal microscope. Scale bars = 2 µm. (**A**) Fluorescence confocal microscopy and fluorescence photorecovery after bleaching (FRAP) analysis of GFP-*KRAS*^G12D^ trafficking within LOVO TNTs. GFP-tagged mutant *KRAS* transfected LOVO cells were co-cultured with CellTracker Red labeled HCT-8 cells; fluorescence confocal microscopy was performed to visualize recovery of fluorescent signals after photobleaching of the area indicated by the area in the rectangle. (**B**) Quantitation of fluorescence photorecovery of the area shown in panel A. The time point of immediate loss of GFP expression occurred at T = 0, after bleaching. GFP fluorescence intensity recovery is graphed as a percentage of initial expression. (**C**) Fluorescence confocal microscopy and FRAP of GFP-*KRAS*^G12D^ visualized at the point of contact of the TNT with the plasma membrane of a recipient HCT-8 cell. The square dotted lines indicate the photobleached region at point of contact. (**D**) Quantitation of fluorescence photorecovery of the area shown in panel (**C**).

To examine whether TNTs redistribute mutant *KRAS*, we co-cultured LOVO cells exogenously expressing green fluorescent protein (GFP)-tagged mutant *KRAS* construct along with HCT-8 cells labeled with CellTracker Red. Using fluorescence microscopy, we confirmed TNT formation and intercellular transport of GFP-tagged mutant *KRAS* within these TNTs (Figure 2A). We next employed fluorescence recovery after photobleaching (FRAP) to quantitate recovery of mutant *KRAS* trafficking within these TNTs (Figure 2B; Video S1).

The recovery time was rapid (within 30–40 s post-photobleaching). However, the extent of this recovery was not 100%. This lack of complete recovery is consistent with other studies using FRAP [15,38,39] and may be due at least in part to an immobile fraction. These data suggest that TNTs are open conduits allowing for transfer of mutant *KRAS* into the recipient cell. In a separate experiment, we further analyzed trafficking of mutant *KRAS* into recipient HCT-8 cells; we performed fluorescence microscopy and FRAP with photobleaching focused on the point of contact of a TNT with the recipient plasma membrane (Figure 2C, Video S2). Quantitative analysis of GFP recovery was again similar to the above experiment (Figure 2D). To ensure that cell trafficking and morphologic cellular changes associated with TNTs were, in fact, due to mutant *KRAS* expression and not attributable to GFP, we examined cells transfected with GFP alone as a negative control and found no changes in cell morphology (Figure S2 upper left panel) This was compared to cells transfected with GFP-tagged mutant *KRAS* (Figure S2 upper right panel) in which there were clear alterations in cell size and development of cell protrusions consistent with TNTs. Additional representative images of GFP-tagged mutant *KRAS* following transfection and protein expression are provided in Figure S2.

### 2.3. Mutant RAS Predominantly Trafficks through rather than along TNTs

**Figure 3:**
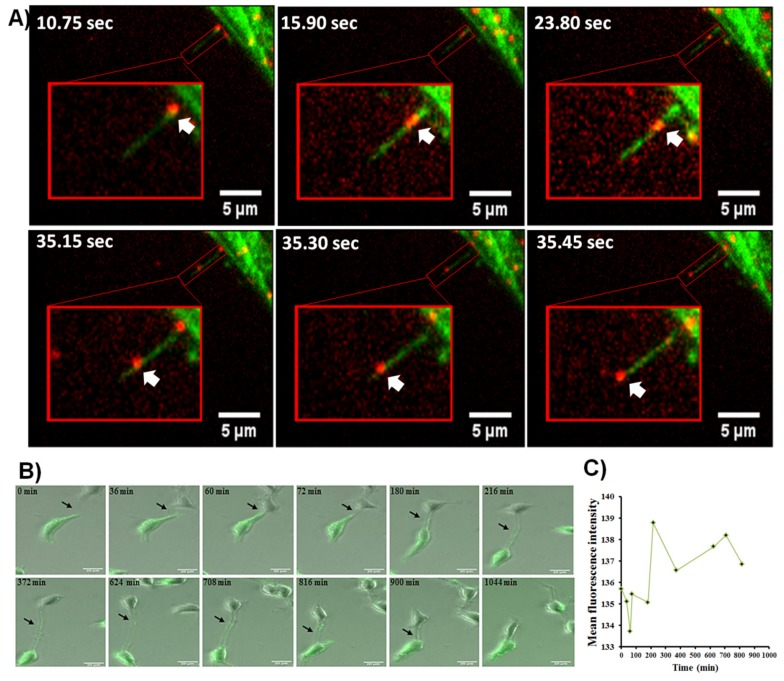
Visualizing intercellular transport of mobile *KRAS* using fluorescence microscopy. (**A**) Fluorescent imaging of two-color JF646-dyed *KRAS*-halo and GFP2 cytoskeleton imaged with the Nikon N-STORM microscope demonstrating RAS motility within actin-based cell protrusions. White arrows indicate actin-based protrusions consistent with TNTs. Scale bars = 5 μm. (**B**) Time-lapse microscopy of a GFP mutant *KRAS*-positive HCT-8 cell using a TNT to transfer mutant *KRAS* to an HCT-8 cell not expressing GFP mutant *KRAS*. Scale bar = 20 μm. (**C**) Graph demonstrating fluctuation of GFP mutant *KRAS* expression within the recipient cell during the indicated time interval. Measurements are reported as mean fluorescence intensity, assessed as corrected total cell fluorescence/area (see Section 4 for details).

The current paradigm of *KRAS* localization as a whole is that the protein is physically connected to the inner leaflet of the plasma membrane. TNTs are extracellular extensions of that plasma membrane and the cell body; thus, membrane-bound isoforms of *KRAS* may not necessarily need to separate from the membrane to traffic through TNTs. In addition, we previously showed that vesicles such as exosomes engulfed by cells can traffic through or along TNTs [23]; it is conceivable that proteins including RAS may traffic through TNTs within endosomal vesicles. To determine whether mutant *KRAS* trafficks through or along TNTs, we examined the *KRAS*4b isoform because this isoform mainly localizes to the plasma membrane whereas the 4a isoform is predominant in the cytoplasm [40]. We utilized HeLa cells as a model system, and confirmed the presence of TNTs using confocal fluorescent and TIRF-based microscopy using GFP-*KRAS*^G12D^-expressing HeLa cells. TNTs formed above the culture glass surface at approximately 3.5 µm, out of range of the evanescent wavefront (Figure S3). Atomic force microscopy (AFM) further elucidated vesicular bulges characteristic of cellular cargo in transit, and also the rough texture of TNT ultrastructure. In particular, the base of the TNTs displayed a series of smaller protrusions that we postulate act as a scaffold that supports the suspension of these non-adherent protrusions, similar to recent reports on membrane tethers using AFM and TNTs using cryo-electron microscopy (Figure S3) [41,42]. We then used JF 646 tagged Halo-*KRAS*4b (N-terminal) and GFP2-cytoskeleton dual labelled HeLa cells to specifically examine *KRAS*4b movement. We found that *KRAS* isoform 4b was in fact in constant motion inside the cell and through actin-based protrusions consistent with TNTs. In the accompanying images, the fluorescent *KRAS* isoform is moving away from the HeLa cell (Figure 3A, Video S3). This finding supports the notion that RAS protein is mobile within the cell, away from the plasma membrane, and that this mobility extends outside of the cell body via cytoplasmic protrusions such as TNTs.

### 2.4. Non-Membrane Bound KRAS Is Mobile and Can Be Trafficked between Cells via TNTs

To further examine whether TNTs contribute to *KRAS* heterogeneity between cells by redistributing mutant *KRAS* to wild-type *KRAS* tumor cells, we co-cultured HCT-8 cells transfected with mutant *KRAS* and non-transfected HCT-8 cells. We then performed fluorescence time-lapse microscopy during a 24-h period. We captured instances of TNT formation between cells and measured direct transmission of GFP-tagged mutant *KRAS* from cell-to-cell (Figure 3B, Video S4). Using Image J software, we measured GFP fluorescence intensity contained within the recipient cell in each image over time (Figure 3C); the fluorescence at each time point was a surrogate measure for concentration of mutant *KRAS* protein co-expressed with the GFP. We found that the overall intensity of GFP-*KRAS* increased during 15 h; however, the increase was not linear. There were spikes in fluorescence due to dynamic transfer of the GFP-*KRAS* through the TNT, with points of lower intensity reflecting the transient nature of this transfer. These findings indicate that mutant *KRAS* promotes TNT formation between cells and that formation of these TNTs facilitates the direct intercellular transfer of mutant *KRAS*.

### 2.5. Mutant KRAS Is Transferred from More Aggressive to Less Aggressive CRC Cells

**Figure 4:**
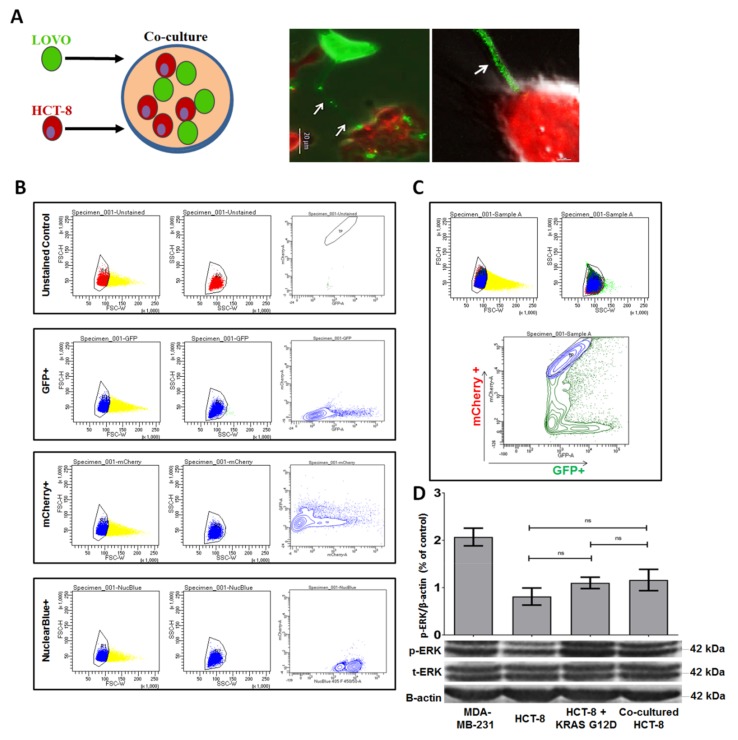
Transfer of mutant *KRAS* from LOVO cells to HCT-8 cells increases ERK phosphorylation and TNT formation in HCT-8 cells. (**A**) Schematic of the LOVO and HCT-8 cell co-culture. LOVO cells transfected with GFP-tagged mutant *KRAS*^G12D^ were co-cultured with HCT-8 cells labeled with Nuclear Blue and mCherry red fluorescent protein. (**B**) FACS-based analysis of mutant *KRAS* transfer between the two cell lines. The four sets of dot plots depict live-cell, single-cell gating for each possible cell population. (**C**) Flow cytometric scatter dot plots of triple-positive HCT-8 cells. Live single events were selected for sorting based on high forward scatter (FSC) and side scatter (SSC) intensity values. Extracted HCT-8 cells positive for GFP, mCherry, and NucBlue were post-sorted to determine the purity of this population. The mixed triple-positive population represented 2–4% of the total sorted cell population. (**D**) Quantitative Western blot analysis of ERK phosphorylation in HCT-8 cells to examine the effects of mutant *KRAS* acquisition. The MDA-231 cell line was used as a positive control for high ERK activity. (HCT-8 + *KRAS* G12D vs. HCT-8: *p*-value = 0.4303; Co-cultured HCT-8 vs HCT-8: *p*-value = 0.4749; Co-cultured HCT-8 vs. HCT-8 + *KRAS* G12D: *p*-value = 0.7414).

After establishing that TNTs are involved in intercellular trafficking of mutant *KRAS*, we sought to determine whether the transfer of mutant *KRAS* to wild-type *KRAS* CRC cells can induce a lasting functional effect by dysregulating downstream molecular signaling in recipient cells. First, we co-cultured LOVO cells transfected with GFP-tagged *KRAS*^G12D^ construct with HCT-8 cells transfected with NucBlue nuclear stain and mCherry red fluorescent protein in a 1:1 ratio for 48 hours (Figure 4A, left panel).

Fluorescence microscopy revealed TNT-mediated interaction between the two cell populations (Figure 4A, middle and right panels). After 48 hours in co-culture, we separated triple-positive (GFP, NucBlue, mCherry) HCT-8 cells by fluorescence-activated cell sorting (FACS). The nuclear marker NucBlue was used to distinguish cells that transferred *KRAS* versus those that received it. FACS analysis demonstrated that transfer of mutant *KRAS* occurred, with the mixed triple-positive population representing 2–4% of the total sorted cell population (Figure 4B,C).

In separate co-culture experiments, blockade of TNTs with or without an actin-destabilizing agent between LOVO and HCT-8 cells reduced the triple-positive population to approximately 1.2% and 0.1%, respectively (Figure S4). Western blot analysis confirmed harvested HCT-8 cells acquired G12D *KRAS* protein during co-culture (Figure S5). Taken together, these experiments demonstrate that cells expressing mutant *KRAS* can transfer mutant *KRAS* to wild-type *KRAS* CRC cells and that this transfer is reduced when TNT formation between the two cell populations is diminished physically and pharmacologically.

### 2.6. Intercellular Acquisition of Mutant KRAS Increases ERK Phosphorylation in Recipient Wild-Type CRC Cells

Mutated RAS regulates the MAPK signaling cascade that induces ERK activation, in part via hyperphosphorylation. We tested whether intercellular transfer of mutant *KRAS* results in upregulation of ERK phosphorylation in recipient cells that express wild-type RAS. We examined sorted HCT-8 cells after co-culture with *KRAS* mutant LOVO cells, HCT-8 cells cultured alone, HCT-8 cells directly transfected with *KRAS*^G12D^ construct, and MDA-MB-231 cells. Acquisition of mutant *KRAS* via coculture with LOVO cells elicited a 35% increase in ERK phosphorylation as compared to HCT-8 cells cultured alone (Figure 4D; full western blot and densitometry readings are provided in Figure S6). The protein expression in HCT-8 cells that acquired G12D after co-culture was higher than protein expression in HCT-8 cells transfected directly with GFP-*KRAS* G12D. The reason for this difference is that the latter group was analyzed post-transfection without cell sorting, and the transfection efficiency was not 100%. The former group was a FACS-sorted (purified) cell population post-transfection; and thus, it demonstrated higher expression.

### 2.7. Mutant KRAS Increases TNT Formation and Reduces Cell Size in Recipient Wild-Type KRAS Cells

**Figure 5:**
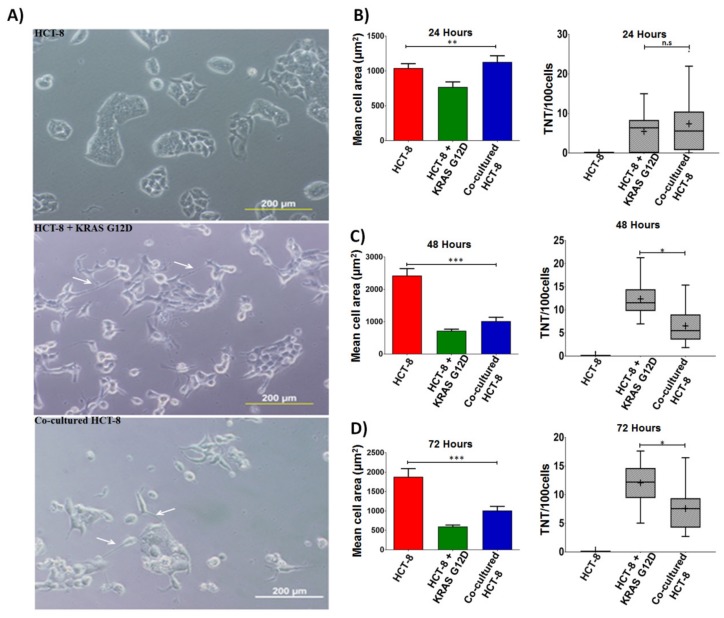
Effects of acquisition vs intercellular transfer of mutant *KRAS* on cell area and TNT formation in recipient colon cancer cells. (**A**) Representative phase contrast microscopy of native HCT-8 cells, HCT-8 cells post-transfection with *KRAS* G12D, and HCT-8 cells co-cultured with mutant G12D-expressing cells. Cells were examined at 24 h intervals to measure cell size and TNT formation. Changes in mean HCT-8 cell size are shown in the left-hand panels at (**B**) 24 h, (**C**) 48 h, and (**D**) 72 h after transfection or co-culture. Boxplot representation of TNTs per 100 HCT-8 cells is shown in the right-hand panels at (**B**) 24 h, (**C**) 48 h, and (**D**) 72 h. Asterisks indicate statistical significance with *p*-value < 0.05 (see Statistical Analysis in the Section 4 for details); n.s. = not statistically significant (*p* > 0.05); per GraphPad Prism standards, * indicates *p* ≤ 0.05; ** indicates *p* ≤ 0.01; *** indicates *p* ≤ 0.001.

To test whether acquisition of mutant *KRAS* affects TNT formation potential, HCT-8 cells were cultured alone, directly transfected with *KRAS* G12D construct, or co-cultured with mutant G12D-expressing LOVO cells (Figure 5A). We first quantified the area of HCT-8 cells and found a decrease in cell area by 48 and 72 h, but not by 24 h (Figure 5B–D); this finding suggests that the effect of mutant *KRAS* transfer on cell morphology was not immediate. We then determined the rate of TNT formation after acquisition of oncogenic *KRAS* at 24, 48, and 72 h.

**Figure 6:**
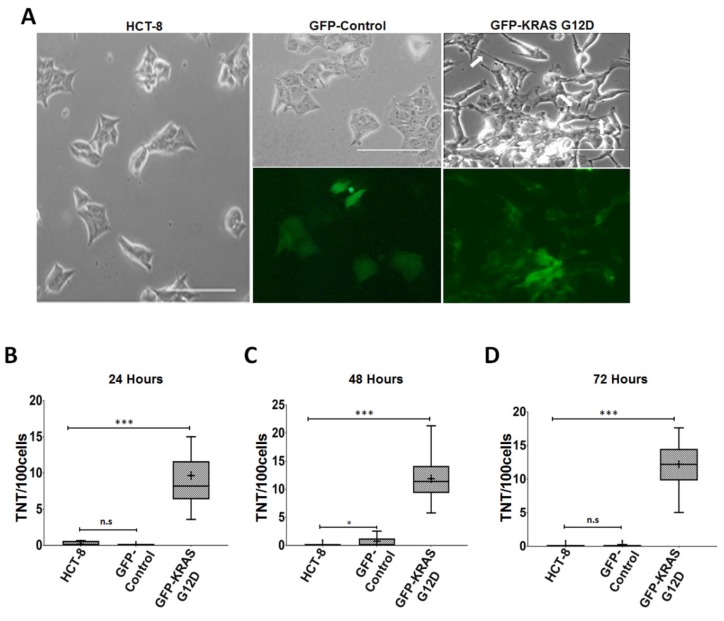
Mutant *KRAS* is associated with higher TNT formation among HCT-8 cells. (**A**) Representative phase contrast and fluorescence microscopy images of HCT-8 cells, HCT-8 cells transfected with GFP-control plasmid, and HCT-8 cells transfected with GFP-*KRAS*^G12D^ mutant. (**B**–**D**) Boxplot representation of TNTs per 100 cells over (**B**) 24 h, (**C**) 48 h, and (**D**) 72 h. Quantification of TNTs was performed using methods similar to Figure 1. Symbols on the boxplot are as follows: Box, 1st to 3rd (Q1–Q3) Quartiles; + = Mean; Line inside box = Median. Asterisk symbol indicates statistical differences in median value; n.s = non statistically significant.

Acquisition of G12D *KRAS* by either transfection or co-culture significantly upregulated TNT formation in recipient HCT-8 cells at all three time points (Figure 5B–D). The difference between transfected vs. co-cultured cells compared to baseline was significant at 48 and 72 h (Table 2). These findings demonstrate that transfer of mutant *KRAS* results in changes in cell morphology, including TNT formation. To further confirm that acquisition of mutant *KRAS* affects TNT formation, we transfected HCT-8 cells with a plasmid expressing the G12D variant of *KRAS* co-expressed with green fluorescent protein (GFP). As a negative control, we separately transfected HCT-8 cells with a GFP-plasmid. We noted morphologic differences in the cells transfected with *KRAS*^G12D^ (Figure 6A). HCT-8 cells that had acquired G12D *KRAS* displayed an altered morphology more characteristic of mesenchymal or fibroblast cells (i.e., more elongated, linear, and spindle-like than epithelial cells) as compared to native HCT-8 cells. HCT-8 cells transfected with G12D *KRAS* also formed significantly more TNTs per 100 cells than non-transfected cells or the GFP control at 24, 48, and 72 h (Figure 6B–D).

## 3. Discussion

Horizontal transmission of oncogenes, and oncogenic proteins, is a relatively novel topic of study in the larger field of intercellular communication in cancer that merits further evaluation for its potential role in creating highly heterogeneous tumor microenvironments. Intercellular transfer of oncogenic RAS has been examined in a few studies, with this transport occurring via extracellular vesicles [12,13] and even TNTs connecting immune cells [15,16,37]. The downstream effects of this transfer, and the role of TNTs in mediating this process in colon cancer particularly, have hitherto been unclear. Our study builds upon prior studies by examining the ability of TNTs to mediate intercellular transfer of mutant *KRAS* protein in colon cancer cells with endogenous wild-type *KRAS*. A schematic depicting this process is provided in Figure S7.

We recently reported that TNTs can be imaged in CRC resected from human patients [25]. Our present study assesses TNT formation in multiple CRC cell lines with various invasive potential in vitro. Our data demonstrate that mutant *KRAS* can be transported between CRC cells and that TNTs represent one mechanism for facilitating direct cell-to-cell transfer between mutant *KRAS* and wild-type *KRAS* CRC cells. Further, the acquisition of mutant *KRAS* by wild-type *KRAS* CRC cells accelerated the rate of TNT formation and activated downstream molecular signaling in the RAS pathway. The transfer of RAS along the filamentous actin, which provides the structural basis of TNTs, is not surprising because it has been shown that high levels of activated RAS trigger cellular protrusions [43]. In that study, it was reported that RAS activation leading to extension of protrusions induces a positive feedback loop. This finding is consistent with our results that *KRAS* mutant CRC cells have upregulated TNT formation, and in turn, these TNTs facilitate intercellular transfer of mutant *KRAS*. In addition, we co-cultured GFP-tagged mutant *KRAS*-expressing CRC cells with wild-type *KRAS* CRC cells (labeled red). GFP-mutant *KRAS* fluorescence was detected for at least 7 days after the start of co-culture. This transfer also led to changes in cell morphology, including decreases in mean cell area, suggesting sustained cellular changes. One remarkable morphological change occurred when acquisition of mutant *KRAS* stimulated a quantifiable increase in the average number of TNTs per cell in wild-type cells. The results suggested that intercellular transfer of mutant *KRAS* led to changes in cell phenotype that were associated with increased TNT-mediated intercellular communication. Most notably, the higher rate of TNT formation among these cells was not dependent on cellular proliferation. 

Our findings suggest that there is significant heterogeneity of TNT formation among CRC cell types. The malignant cell lines LOVO and HCT-116 both harbor *KRAS* G13D and formed the most TNTs. LOVO cells formed significantly more TNTs over time than HCT-116. We noted that LOVO is derived from a metastatic CRC tumor, and HCT-116 from a primary colon tumor; the metastatic phenotype is more invasive, and this property may account for this difference. However, the wild-type malignant cell line HCT-8 also formed a small but nonetheless quantifiable amount of TNTs. In examining other common genetic factors among these three CRC cell lines forming TNTs (LOVO, HCT-116, HCT-8), we noted that each of these cell lines is also marked by deficiency of mismatch repair proteins (dMMR), a genetic factor that can lead to microsatellite instability (MSI) [30,33,34]. Approximately 45% of MSI/dMMR cases also harbor mutations in *KRAS* [44,45,46]. Among our three CRC cell lines forming TNTs, LOVO cells harbor MSI and G13D *KRAS* and are derived from a metastatic lesion; this cell line formed significantly more TNTs than HCT-116 and HCT-8; HCT-116 is characterized by MSI and G13D *KRAS*, but derived from a primary tumor, and HCT-8 is characterized by only MSI. Therefore, metastatic colon cancer cells harboring the combination of mutant *KRAS* along with MSI displayed a higher potential for forming TNTs, a highly relevant finding in light of recent evidence that MSI-high tumors are more strongly susceptible to immune checkpoint inhibitor therapy than microsatellite stable (MSS) tumors [47].

The molecular drivers and genomics associated with CRCs have been better elucidated in recent years, including significant characterization provided by The Cancer Genome Atlas Network (TCGA) [48]. Recent advances in the molecular classification of CRCs are highly clinically relevant, with more widespread adoption of next-generation sequencing and All-RAS testing to identify additional isoforms of *KRAS* and *NRAS*. CRC tumors were previously classified mainly into several molecular subtypes that included chromosomal instability, microsatellite instability, and DNA hypermethylation or CpG island methylator phenotype (CIMP) [49]. Stratification currently aligns into four molecular subgroups that are now well described and prominent for their correlation to patient outcomes, including survival [50]. Our study provides insight into the notion that the presence of mutated *KRAS*, as well as MSI-H tumor status, are associated with increased formation of TNTs. MSI-H CRC cell lines that are commonly used for cancer research have been associated with proximal tumor location (a factor in turn recently correlated with worse prognosis in CRC patients), as well as CIMP-positive status [32]. CRC tumors with positive CIMP, in particular, may in turn involve cytoskeletal remodeling regulated by key RAS-associated proteins, such as oncogenic RASSF1A (Ras-association domain family isoform), a tumor suppressor whose loss has been recently implicated in TNT formation [51]. This combination of molecular and cellular alterations undoubtedly influences the evolving tumor microenvironment, both prior to and following cancer-directed treatment of such tumors. 

RAS regulates MAP-kinase signaling pathway that relays information from the surface of the cell to the nucleus by way of Ras/Raf/MEK/ERK phosphorylation [52]. The functional transfer of mutant *KRAS* was demonstrated by a 35% increase in the phosphorylation of ERK in *KRAS* wild-type cells after transfer of oncogenic *KRAS.* The long-term implications of this downstream effect are yet unknown; recent studies evaluating transfer of oncogenic H-ras via extracellular vesicles from transfected intestinal (non-malignant) epithelial cells detected an ability of this transfer to regulate recipient endothelial cells, but not to induce malignant transformation [13]. The authors of that study suggested that despite this fact, malignant cells preferentially received and were affected by these Ras-carrying vesicles, and that finding is consistent with our observation in this study detecting differences in downstream molecular regulation of the RAS pathway in recipient colon cancer cells. A separate and more recent study showed that intercellular transfer of *KRAS* via extracellular vesicles failed to induce any meaningful effects in recipient cells [53]; thus, it is clear that with relatively disparate results across cell types, and even using different members of the RAS family (*KRAS* and *HRAS*), these cumulative studies performed to date raise important questions regarding efficiency of transfer, whether a minimum amount of transported RAS cargo is required to initiate short- or long-term alterations in recipient cells, and whether cellular and molecular properties are altered in malignant cells alone as compared to non-malignant cells.

It is well known that the effects of oncogenic mutant *KRAS* can also be mediated by noncanonical signals via multiple pathways [54], and therefore, the gene transfer can be inferred to have pleiotropic effects beyond MAPK signaling. These findings have implications well beyond just colon cancer, as indeed transfer of oncogenic RAS may be a newly identified hallmark of many other types of RAS-driven cancers. Mueller et al. recently reported that allelic imbalance and increased oncogenic dose of RAS increases metastatic potential in pancreatic carcinomas [55], and the intercellular transfer of oncogenic RAS may be one modality by which this imbalance is achieved. These findings also have important implications for fully understanding intercellular communication in cancer. Intriguingly, increased expression of the RAS oncogene product has been associated with downregulation of gap junction intercellular communication (GJIC) [56,57,58,59]. As GJIC occurs between cells in immediate proximity, the downregulation and abrogation of these connexin channel-lined junctions may lead to increased intercellular cross-talk mediated by TNTs among RAS-expressing cells. 

It is well established that *KRAS* signaling induces changes in the extracellular matrix that are highly conducive to tumor expansion and metastasis [60]. Our finding that CRC cells transfer a tumor-driving mutant molecule to other cells is consistent with previous observations that TNTs formed between immune-related cells [15]. *KRAS* is present in recycling endosomes, but localizes to the plasma membrane when active [61]. Reports of RAS transfer have involved the sharing of membrane patches, similar to the process known as trogocytosis [62], as well as intercellular transport of oncogenic RAS via extracellular vesicles [12,13]. 

The results we present here suggested that oncogenic mutant *KRAS* increases TNT-mediated cross-talk between colon cancer cells and that these cells harness these TNTs to further disperse *KRAS* molecules to other wild-type *KRAS* colon cancer cells. Demonstrating horizontal transfer of *KRAS* represents a highly novel and transformative shift in the current paradigm that driving mutations only occur either through vertical transmission or come about spontaneously through mutation. This is a finding that may explain how proportions of mutant subclones can be dynamic over time and with tumoral evolution and also why mutant RAS localizes to regions of highest invasive potential in CRC [8].

## 4. Materials and Methods 

### 4.1. Cell Lines and Cell Culture

LOVO, HCT 116, SW480, and HT-29 cell lines were acquired from the American Type Culture Collection (ATCC, Rockville, MD, USA). The colon cancer HCT-8 cell line was kindly provided by Dr. Timothy Starr at the University of Minnesota. The AAC1 adenoma cell line was provided by Dr. Subbaya Subramanian at the University of Minnesota. The LOVO cell line is a human G13D *KRAS* mutant colon cancer cell line derived from a distant (supraclavicular) metastasis [26]. HCT 116 is a human G13D *KRAS* mutant colon carcinoma derived from a primary tumor [26]. SW480 is a G12V *KRAS* mutant colon cancer cell line derived from a primary tumor [63]. HCT-8 and HT-29 are *KRAS* wild-type human colon carcinoma cell lines derived from primary tumors [26,30]. AAC1 is a non-malignant, *KRAS* wild-type human colon adenoma cell line [64]. Three of these cell lines have also been identified to exhibit microsatellite instability (LOVO, HCT 116, HCT-8). Authentication of cell lines was performed by the Core Fragment Analysis Facility at Johns Hopkins University using short tandem repeat (STR) profiling. Cell lines were confirmed to be negative for mycoplasma contamination within six months of use. Please see Table 1 (in Section 2) for further details.

LOVO cells were maintained and passed using Ham’s F-12 medium supplemented with 10% FCS, vitamins, antibiotics, and glutamine; HCT-8 and HCT116 cells were maintained in Dulbecco’s modified Eagle’s medium (DMEM) supplemented with 10% fetal bovine serum (FBS; Invitrogen Life Technologies, Carlsbad, CA, USA), and 1% Antibiotic-Antimycotic (anti-anti; Gibco Life Technologies, Gaithersburg, MD, USA). All cell lines were incubated in a humidified incubator at 37 °C supplied with 5% carbon dioxide.

### 4.2. Transfections

For transfections, RAS expression clones CMV51p > mCherry-Hs.*KRAS* and CMV51p > eGFP-Hs.*KRAS*^G12D^ were obtained from the RAS Program at the NCI’s Frederick National Laboratory for Cancer Research (FNLCR, Frederick, MD, USA). This clone specifically co-expressed GFP with the G12D variant of *KRAS*. Cell lines were grown in passaging medium without antibiotics to 70–90% confluence at the time of transfection. Transfection was performed 24 hours after plating using Lipofectamine 2000 (Invitrogen, Carlsbad, CA, USA) and 2 μg of plasmid DNA in Opti-MEM^®^ without serum following the manufacturer’s instructions. Cells were used in experiments 24–60 h post-transfection. For co-cultures, LOVO cells transfected with GFP-tagged *KRAS*^G12D^ and mCherry and Pure Blu Nuclear Staining Dye double-labelled HCT-8 cells were cultured in a 1:1 ratio. Cells were left in culture for 48 hours after which HCT-8 cells were acquired through fluorescence-activated cell sorting and media selection.

### 4.3. Quantification of TNTs

TNTs were visually identified and quantified as previously described [9,20,65,66,67]. Briefly, these parameters included: (i) lack of adherence to the substratum of tissue culture plates, including visualization of TNTs passing over adherent cells; (ii) TNTs connecting two cells or extending from one cell were counted if the width of the extension was estimated to be < 1000 nm; and (iii) a narrow base at the site of extrusion from the plasma membrane. Cellular extensions not clearly consistent with the above parameters were excluded. Images for TNT analysis were captured on an Olympus IX70 inverted microscope (Olympus Corporation, Center Valley, PA, USA) using the 20 × objective lens in 15 randomly chosen fields of a 6-well plate at 24, 48, and 72 h. A single representative image taken at all time points for each well was used for analysis of TNT rate, length, and cell proliferation. Experiments were performed in triplicates for each cell line. The number of TNTs per cell (TNTs/cell) was used to represent our findings to exclude the possibility that changes in TNTs were due to increased cell proliferation. ImageJ was used to convert images to 32-bit to correct for color discrepancies. Cells and TNTs were counted manually. Box plots were generated to show the distribution of the data, and medians were compared using the Mann Whitney *U* test assuming unequal variances. *p*-values < 0.05 were considered statistically significant.

### 4.4. FACS Sorting

Co-cultured cell samples were harvested and filtered to ensure single-cell suspension in 5 mL polystyrene test tubes (Falcon, Corning, NY, USA). The final sample was resuspended in RPMI-1640 medium supplemented with 2.5% FBS. Cell populations of interest were acquired and sorted with a FACSAria II cell sorter (BD Biosciences, Franklin Lakes, NJ, USA) equipped with a 488 nm laser (50 mW Sapphire Laser; Coherent Inc, Santa Clara, CA, USA), 561 nm laser (100 mW Sapphire Laser, Coherent Inc., Santa Clara, CA, USA), and 405 nm laser (100 mW OBIS Laser, Coherent Inc., Santa Clara, CA, USA) for GFP, mCherry, and NucBlue excitation, respectively. Using FACSDiva software (BD Biosciences), HCT-8 single-cell events were distinguished and sorted from the population by gating for GFP, mCherry, and NucBlue triple-positive cells with similar FSC/SSC characteristics as the HCT-8 negative control.

### 4.5. Time-Lapse Imaging

For time-lapse imaging of mutant *KRAS*-positive TNTs, cells (LOVO or HCT-8) were transfected with GFP-*KRAS*^G12D^ in a ratio of 1:1 and at a density of 3 × 10^4^ cells/35 mm dish. After 4 h, the dishes were evaluated to ensure even distribution. After 24 h, the dishes were scanned for GFP-*KRAS*^G12D^ and negative cells that were in proximity and could potentially interact via TNTs. These regions of interest were imaged for generative time-lapse movies. In brief, fields of view through a 20 × objective lens were captured using a wide-field Axio200M microscope (Zeiss, Inc., Thornwood, NY, USA) custom-fitted with a stage incubator that maintains environmental conditions at 37 °C and 5% CO_2_. The microscope was set up to capture an image of each chosen field every 10 s in the differential interference contrast (DIC) and green fluorescent channels for 10 min.

### 4.6. Quantification of Fluorescence after Intercellular Transfer of GFP-Tagged RAS

Changes in GFP fluorescence intensity within the recipient cell were measured using ImageJ software. The cell of interest was selected using the drawing/selection tools (circle tool). From the “Analyze” menu of the software, “set measurements” was used to select “area integrated intensity” and “mean grey value.” The GFP fluorescence intensity value was measured for both the cell of interest and the image backgrounds. The data from the “results” window was transferred into a new spreadsheet (MS Excel) and the corrected total cell fluorescence (CTCF) formula was used: CTCF = Integrated Density—(Area of selected cell * Mean fluorescence of background readings).

### 4.7. Cell Lysate

Cells were washed quickly with cold PBS (phosphate buffered saline), scraped from culture plates, and then subjected to centrifugation (13,000 rpm, 14 s). The pellets were resuspended in 5 × the pellets’ volume of TNESV lysis buffer (50 mM Tris-HCl, pH 7.4; 1% NP-40; 2 mM EDTA, pH 8.0; 0.1 M NaCl) containing protease (Roche, Indianapolis, IN, USA) and phosphatase inhibitors (Sigma-Aldrich, St. Louis, MO, USA) for 10 min at room temperature. Lysed cells were subjected to centrifugation (13,000 rpm, 14 s), and supernatant was collected. Protein concentrations of lysate were determined by Bradford assay (Bio-Rad, Hercules, CA, USA). 

### 4.8. Western Blots

Proteins were separated by 8 or 10% SDS-polyacrylamide gel electrophoresis (PAGE) gels. Separated proteins were transferred to polyvinylidene difluoride (PVDF) membranes, and the membranes were blocked in 5% non-fat dry milk for 1 hour at room temperature in Tris-buffered saline-Tween (TBST: 0.15 M NaCl; 0.01 M Tris-HCl, pH 7.6; 0.05% Tween 20). PVDF membranes were then blocked for 1 hour at ambient temperature and then overnight at 4 °C with primary antibody. Primary antibodies were acquired from Cell Signaling Technology, Inc. (Danvers, MA, USA) and were rabbit Ras (CST#3965), Ras (G12D Mutant Specific) (SCT #14429), p44/42 MAPK (Erk1/2) (CST #9102), and Phospho-p44/42 MAPK (Erk1/2) (CST #9101). Each antibody was diluted 1:1000 prior to use. Membranes were cut, and each protein of interest was probed separately. Blots were washed three times for 5 min in TBST before and after incubation with the appropriate horseradish peroxidase labeled secondary antibodies. A LI-COR-Odyssey infra-red scanner (LI-COR Biosciences, Lincoln, NE, USA) was used to visualize the bands of interest. Protein band density was measured by using ImageJ (public domain, https://imagej.nih.gov).

### 4.9. Photobleaching (FRAP)

For FRAP experiments, LOVO colon carcinoma cells were transfected with a plasmid expressing fluorescently labeled GFP-*KRAS*G12D (green). After confirmation of successful transfection and fluorescent signal, these cells were co-cultured with HCT-8 colon carcinoma cells labeled with CellTracker Red. FRAP was performed using a 60 × water immerged objective lens of a Nikon A1Rsi confocal microscope (Nikon Instruments Inc., Melville, NY, USA) A section of interest on GFP-*KRAS* mutant-positive TNTs (panel A of corresponding Figure 2) was selected and photobleached. In an additional experiment, the frame focused on a CellTracker Red labeled HCT-8 cell connected to a GFP *KRAS* G12D-expressing LOVO cell, and photobleaching was applied to the point of contact of the TNT with the recipient HCT-8 cell membrane. For both experiments, recovery of the bleached region was monitored and captured though time-lapse image acquisition.

### 4.10. Two-color Total Internal Reflection Fluorescence (TIRF) Imaging of KRAS4b

After culture overnight in a 6-well plate, confluent HeLa cells were dual transfected with Halo-*KRAS*4b and pCMVLifeAct-GFP2 (ibidi) plasmids and cultured 24 h. Transfected cells were then re-plated onto a glass coverslip and cultured overnight. Halo-*KRAS*4b-expressing cells were further labeled with the JF646 dye conjugated with the ligand (25 pM), which directly penetrates the cell membrane and covalently binds halo-*KRAS*4b, by incubating at 37 °C for 30 min and followed by washing with PBS (1×) three times before imaging. 

The two-color *KRAS*4b and cytoskeleton were imaged with a Nikon N-STORM microscope. Halo-*KRAS*4b (tagged with JF646 dye) molecules and the actin cytoskeleton (GFP2) were excited simultaneously by the 647 nm and 488 nm lasers, respectively, under TIRF mode, while the emissions from the two fluorophores were split into the green and far red channels through the Gemini light splitting system (Hamamatsu, Bridgewater, NJ, USA). The two channel signals were recorded in separate areas of the EMCCD camera (DU888, Andor, Concord, MA, USA) at the frame rate of 20 fps. The two-color channel registration was calibrated with 200 nm tetraspeck fluorescent beads (Life Technologies, Carlsbad, CA, USA) before image acquisition. Image processing was performed to combine the two channel images into a color image stack and made into movie.

### 4.11. TIRF Imaging of HeLa Cells

Differential interference contrast (DIC) images were captured using a Zeiss Widefield microscope in TIRF acquisition mode with a Photometrics DV2/camera imaging system. Images were captured with an alpha Plan-Apochromat100 × Oil objective lens with a numerical aperture of 1.46 and 1.6 optovar magnification. Live cells were cultured on a 35 mm Mattek dishes #1.5 (MatTeK Inc., Ashland, MA, USA) within an imaging chamber at 37 °C and 5% CO_2_.

### 4.12. Cell Preparation for Atomic Force Microscopy

HeLa cells were fixed for 10 min in 2.5% glutaraldehyde, then washed three times with PBS. Cells were then kept in PBS for atomic force microscopy imaging in a 50 × 9 mm Petri dish (product number 351006, BD Falcon, Corning, NY, USA). Atomic force microscopy images were obtained using a MFP-3D-BIO Atomic Force Microscope (Oxford Instruments, Abingdon, UK).

### 4.13. Statistical Analysis

The overall median of TNTs/cell and lengths over three days was compared using the Wilcoxon Rank Sum tests due to the non-normal distribution of data. *p*-values were conservatively adjusted for multiple comparisons within each experiment using a Bonferroni correction. Analyses were conducted using GraphPad Prism version 7 (GraphPad Software, Inc., La Jolla, CA, USA), and *p*-values < 0.05 were considered statistically significant. Prism uses the following symbols indicating extent or lack of statistical significance: ns indicates *p* > 0.05; * indicates *p* ≤ 0.05; ** indicates *p* ≤ 0.01; *** indicates *p* ≤ 0.001; **** indicates *p* ≤ 0.0001.

## 5. Conclusions

In conclusion, our investigation of the intercellular transfer of *KRAS* via TNTs has elucidated a novel mechanism by which a tumor-driving mutant protein can be trafficked and distributed among cancer cells, in this case colon cancer cells specifically. This intercellular transfer induces intracellular heterogeneity of mutant *KRAS* in cells with endogenous wild-type *KRAS*. This finding has strong implications for the ability of TNTs and other modes of intercellular transfer to potentially reprogram malignant and stromal cells in the surrounding tumor microenvironment. Furthermore, as oncogenic RAS signaling has profound effects on modulating the tumor microenvironment, including cancer-associated fibroblasts, immunogenic anti-tumor response, and angiogenesis [60], amplification of the influence of oncogenic RAS via intercellular transfer of this oncogene and its products may have important consequences for tumor progression. Effective therapeutic drugs targeting RAS signaling are desperately needed, as the RAS oncogene drives 30% of all cancers [68]. If TNTs mediate this transfer, then therapeutically targeting these structures by preventing formation and/or disassembling of these cellular conduits represents a novel therapeutic strategy for modulating oncogenic signaling.

## Figures and Tables

**Table 1:** Clinical, molecular, and genetic characteristics of cell lines used in this study.

Cell Line Number	Cell Line	Primary Tissue of Origin	Metastasis	*KRAS* Wt or Mutant	Microsatellite Status	BRAF Wt or Mutant	CMS Group	CIMP Status	References
1	LOVO	Colon	Left supraclavicular region	p.G13D	Unstable (MSI-H)	WT	CMS1	CIMP-negative	[26,27,28]
2	HCT-116	Ascending colon	n/a	p.G13D	Unstable (MSI-H)	WT	CMS4	CIMP-positive	[26,27,28]
3	SW480	Descending colon	n/a	p.G12V	Stable	WT	CMS4	CIMP-negative	[27,28,29]
4	HCT-8	Small intestine (ileocecal)/colorectal	n/a	WT	Unstable (MSI-H)	WT	CMS4	unknown	[30]
5	HT-29	Colon	n/a	WT	Stable	p.V600E	CMS3	CIMP-positive	[26,27]
6	AAC1	Colon	n/a	WT	Stable	WT	n/a (adenoma)	n/a	[31]

wt = wild-type; CMS = Consensus Molecular Subtype; CIMP = CpG Island Methylator Phenotype; MSI-H = Microsatellite Instability-High status. Additional pertinent references: [27,28,32].

**Table 2:** Statistical analysis comparing HCT-8 cells either transfected with *KRAS* G12D or co-cultured with *KRAS* G12D-harboring HCT-8 cells, compared to native HCT-8 cells. *t*-test analysis was performed using GraphPad Prism. *p*-values < 0.05 were considered significant.

Figure	Comparison Groups	Cells Examined	*p*-Value
5B (24 h)	HCT-8	HCT-8 + *KRAS* G12D (green bar)	*p* = 0.0080
5B	HCT-8	Co-cultured HCT-8	*p =* 0.4263
5C (48 h)	HCT-8	HCT-8 + *KRAS* G12D (green bar)	*p =* 0.0001
5C	HCT-8	Co-cultured HCT-8	*p* = 0.0001
5D (72 h)	HCT-8	HCT-8 + *KRAS* G12D (green bar)	*p* = 0.0001
5D	HCT-8	Co-cultured HCT-8	*p* = 0.0005

## Supplementary Materials

The following are available online at https://www.mdpi.com/2072-6694/11/7/892/s1: Figure S1: (A) Differences in cellular proliferation for each cell line. (B) Additional representative phase contrast microscopy images of especially long TNTs forming between LOVO cells. Scale bar = 200 µm. Figure S2: Transfection of GFP alone (upper left panel) into LOVO cells does not induce changes in cellular morphology, whereas transfection with GFP-tagged mutant *KRAS* (*KRAS*^G12Vmut^) upregulates formation of TNTs and TNT-like protrusions. Additional representative inverted fluorescence microscopy images of *KRAS*^G12V^-expressing LOVO cells are provided in the bottom panels. Figure S3: (A) Representative TIRF-based microscopy images demonstrating the non-adherent characteristic of TNTs and the technical challenge of for performing single molecule tracking analysis of *KRAS* within TNTs. (B) Cartoon depiction of TIRF microscopy and the inability of the TIRF-generated evanescent field to reach TNTs above the substratum. FRAP analysis demonstrating recovery of GFP-*KRAS*^G12D^. (C) High resolution confocal microscopic image of HeLa cells connected at long range via a TNT. Note characteristic bulges of intra-TNT vesicles in transit on the right half of the TNT, as well as shorter actin-based adherent stress fibers on both cells, providing contrast to the much longer and non-adherent TNT. Scale bar = 2 µm. (D) Atomic force microscopy (AFM) imaging of TNTs connecting HeLa cells further elucidates the ultrastructure of TNTs, including at the point of entry/extrusion from cells. Insets include Phase-Contrast Microscopy version of the same cells for comparison, and close-up views of the base of the TNT and point-of-contact with both cells. Figure S4: FACS-based analysis of mutant *KRAS* transfer between the two cell lines when cell culture medium was treated with (A) or without (B) cytochalasin D (an actin-destabilizing agent that reduces TNT numbers in vitro [69,70]). LOVO and HCT-8 cells were cultured in separated compartments using a semipermeable transwell culture insert with 0.4 μm diameter pores. The insert allows for free diffusion of culture media contents. Figure S5: Western blot analysis using a *KRAS* G12D mutant specific antibody for qualitative demonstration of acquisition of mutant *KRAS* by HCT-8 cells. The full original blot is provided in the lower panel for context. Figure S6: Full western blot and densitometry data for analysis of downstream effects of KRAS transfer on p-ERK. Figure S7: Schematic visualizing the exchange of mutant *KRAS* between CRC cells through TNTs. Video S1: Time-lapse microscopy movie of FRAP-based mutant *KRAS* transport analyzed within a TNT. Video S2: Time-lapse microscopy movie of FRAP-based mutant *KRAS* transfer analyzed at the LOVO TNT and HCT-8 cell membrane interface. Video S3: Time-lapse microscopy movie of JF646 dyed HALO tagged *KRAS* moving within cellular protrusions. Video S4: Time-lapse microscopy movie of HCT-8 cell sharing GFP mutant *KRAS* protein via a TNT.
