# Supplementary material for: Intercellular Transfer of Oncogenic KRAS via Tunneling Nanotubes Introduces Intracellular Mutational Heterogeneity in Colon Cancer Cells
Source: Cancers (Basel). 2019 Jun 26;11(7):892. doi: 10.3390/cancers11070892 (PMC6678395; doi:10.3390/cancers11070892)
Supplement: Supplementary file 1 [file cancers-11-00892-s001.zip › supplementary/cancers-517983-supp-final.pdf]

Supplementary Materials: Intercellular Transfer of Oncogenic KRAS via Tunneling Nanotubes Introduces Intracellular Mutational Heterogeneity in Colon Cancer Cells

Snider Desir, Phillip Wong, Thomas Turbyville, De Chen, Mihir Shetty, Christopher Clark, Edward Zhai, Yevgeniy Romin, Katia Manova-Todorova, Timothy K Starr, Dwight V. Nissley, Clifford J Steer, Subbaya Subramanian and Emil Lou

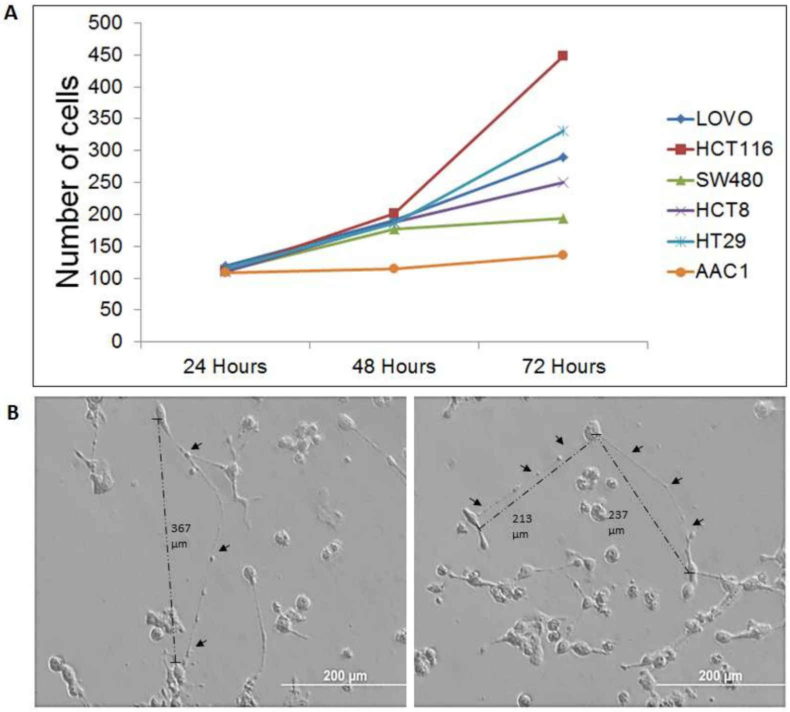

Figure S1. (**A**) Differences in cellular proliferation for each cell line. (**B**) Additional representative phase contrast microscopy images of especially long TNTs forming between LOVO cells. Scale bar = 200 µm.

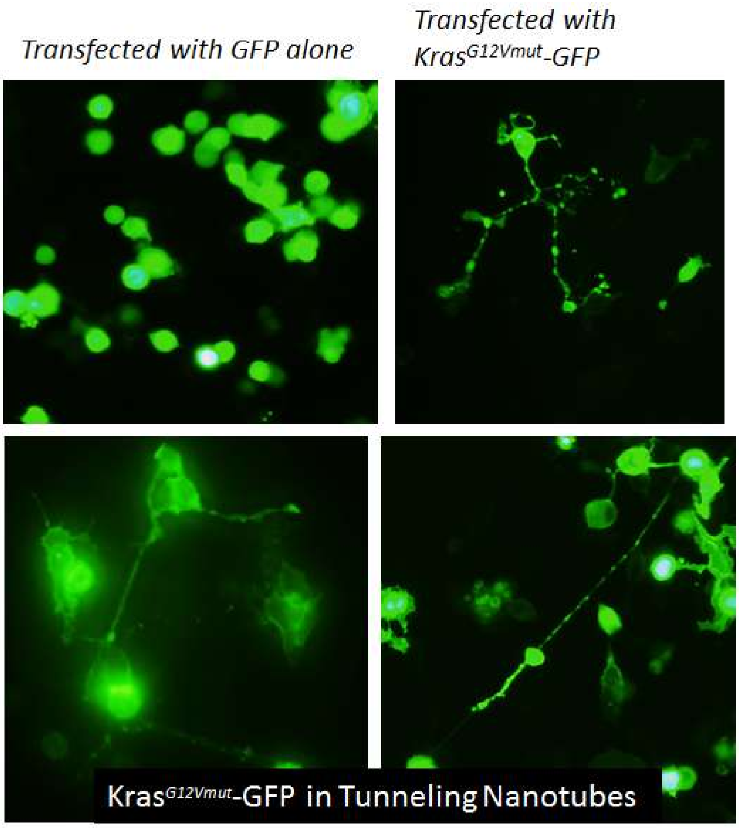

Figure S2. Transfection of GFP alone (upper left panel) into LOVO cells does not induce changes in cellular morphology, whereas transfection with GFP-tagged mutant KRAS (Kras^{G12Vmut}) upregulates formation of TNTs and TNT-like protrusions. Additional representative inverted fluorescence microscopy images of KRAS^{G12V}-expressing LOVO cells are provided in the bottom panels.

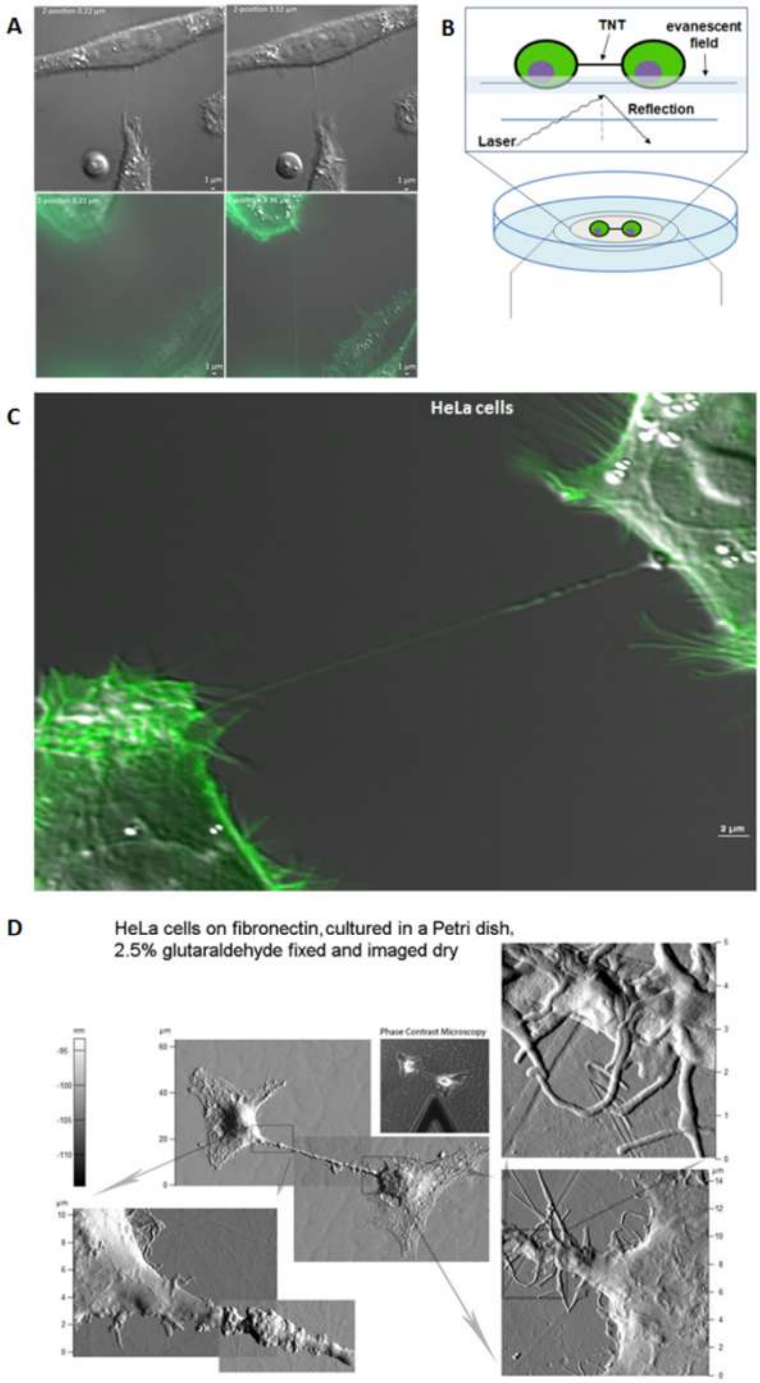

Figure S3. (**A**) Representative TIRF-based microscopy images demonstrating the non-adherent characteristic of TNTs and the technical challenge of for performing single molecule tracking analysis of KRAS within TNTs. (**B**) Cartoon depiction of TIRF microscopy and the inability of the TIRF-generated evanescent field to reach TNTs above the substratum. FRAP analysis demonstrating recovery of GFP-KRAS^{G12D}. (**C**) High resolution confocal microscopic image of HeLa cells connected at long range via a TNT. Note characteristic bulges of intra-TNT vesicles in transit on the right half of the TNT, as well as shorter actin-based adherent stress fibers on both cells, providing contrast to the much longer and non-adherent TNT. Scale bar = 2 μm. **(D)** Atomic force microscopy (AFM) imaging of TNTs connecting HeLa cells further elucidates the ultrastructure of TNTs, including at the point of entry/extrusion from cells. Insets include Phase-Contrast Microscopy version of the same cells for comparison, and close-up views of the base of the TNT and point-of-contact with both cells.

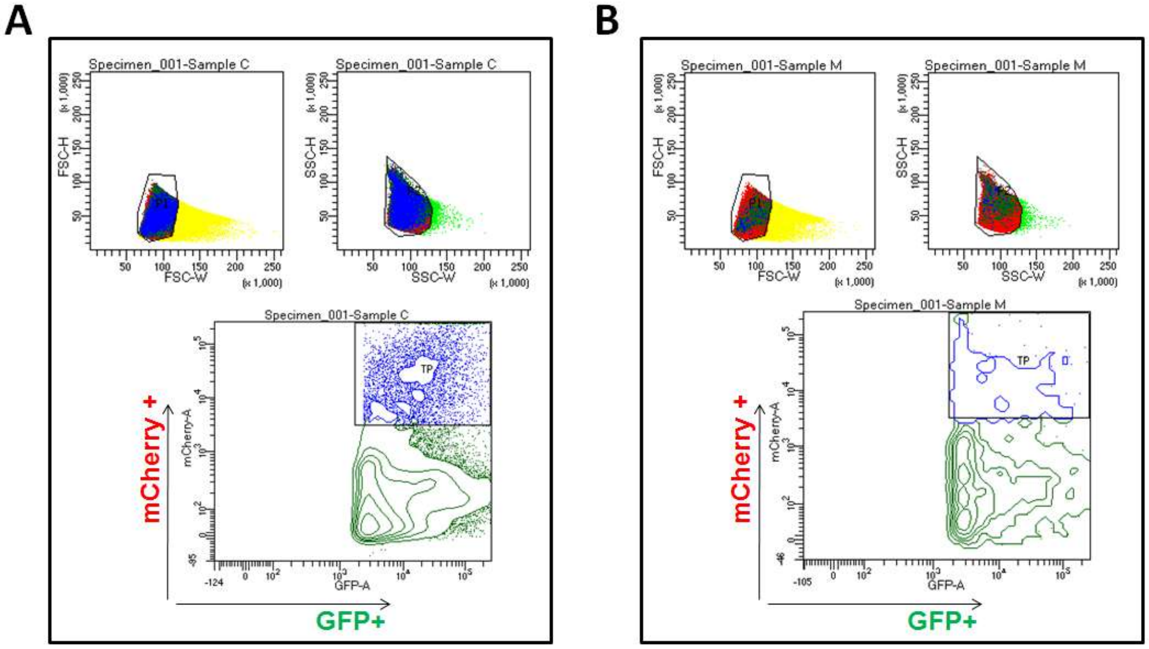

Figure S4. FACS-based analysis of mutant KRAS transfer between the two cell lines when cell culture medium was treated with (**A**) or without (**B**) cytochalasin D (an actin-destabilizing agent that reduces TNT numbers in vitro [69,70]). LOVO and HCT-8 cells were cultured in separated compartments using a semipermeable transwell culture insert with 0.4 μm diameter pores. The insert allows for free diffusion of culture media contents**.**

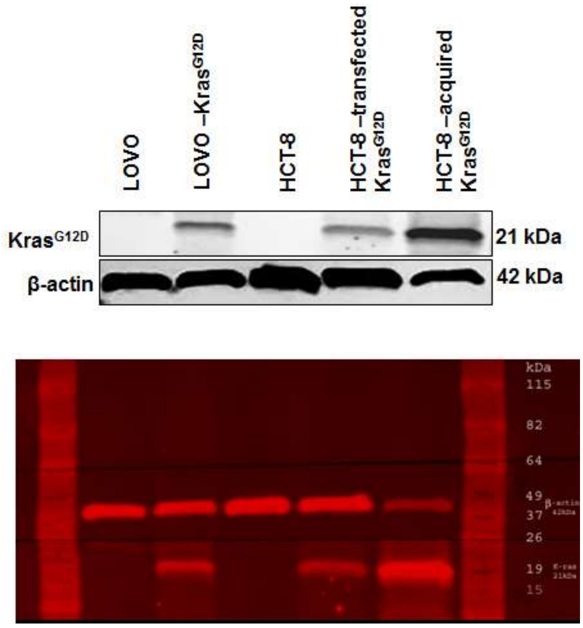

Figure S5. Western blot analysis using a KRAS G12D mutant specific antibody for qualitative demonstration of acquisition of mutant KRAS by HCT-8 cells. The full original blot is provided in the lower panel for context.

Table Analyzed	24	Table Analyzed	24	Table Analyzed	24
Column B	2	Column C	3	Column D	4
vs.	vs.	vs.	vs.	vs.	vs.
Column A	1	Column A	1	Column A	1
Paired t test		Paired t test		Paired t test	
P value	0.0179	P value	0.0817	P value	0.1094
P value summary	*	P value summary	ns	P value summary	ns
Significantly different (P < 0.05)?	Yes	Significantly different (P < 0.05)?	No	Significantly different (P < 0.05)?	No
One- or two-tailed P value?	Two-tailed	One- or two-tailed P value?	Two-tailed	One- or two-tailed P value?	Two-tailed
t, df	t=7.373 df=2	t, df	t=3.28 df=2	t, df	t=2.77 df=2
Number of pairs	3	Number of pairs	3	Number of pairs	3
How big is the difference?		How big is the difference?		How big is the difference?	
Mean of differences	-1.258	Mean of differences	-0.9702	Mean of differences	-0.9091
SD of differences	0.2955	SD of differences	0.5124	SD of differences	0.5686
SEM of differences	0.1706	SEM of differences	0.2958	SEM of differences	0.3283
95% confidence interval	-1.992 to -0.5239	95% confidence interval	-2.243 to 0.3026	95% confidence interval	-2.322 to 0.5032
R squared (partial eta squared)	0.9645	R squared (partial eta squared)	0.8432	R squared (partial eta squared)	0.7932
How effective was the pairing?		How effective was the pairing?		How effective was the pairing?	
Correlation coefficient (r)	0.5682	Correlation coefficient (r)	-0.853	Correlation coefficient (r)	-0.2596
P value (one tailed)	0.3077	P value (one tailed)	0.1748	P value (one tailed)	0.4164
P value summary	ns	P value summary	ns	P value summary	ns
Was the pairing significantly effective?	No	Was the pairing significantly effective?	No	Was the pairing significantly effective?	No

p-ERK assessment by Western Blot					
		MDA-MB-231	HCT-8	HCT-8 + KRAS G12D	Co-cultured HCT-8
	Lanes	1	2	3	4
	Densitometry readings	2.317966	1.169359	0.870609	0.757016
		2.188413	0.59583	1.1537	1.53712
		1.703672	0.670922	1.275013	1.188471

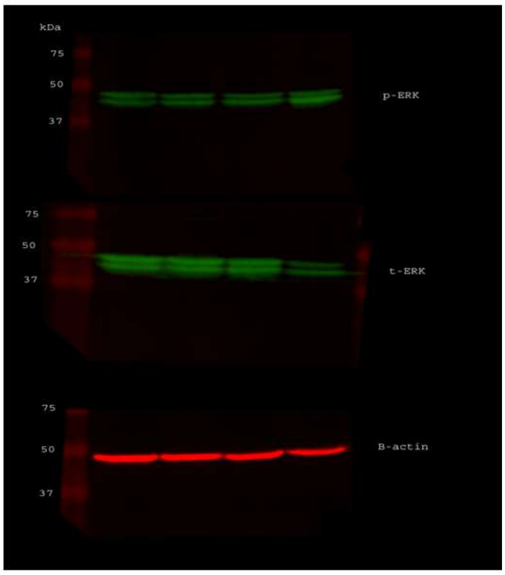

Figure S6. Full Western blot and densitometry data for analysis of downstream effects of KRAS transfer on p-ERK.

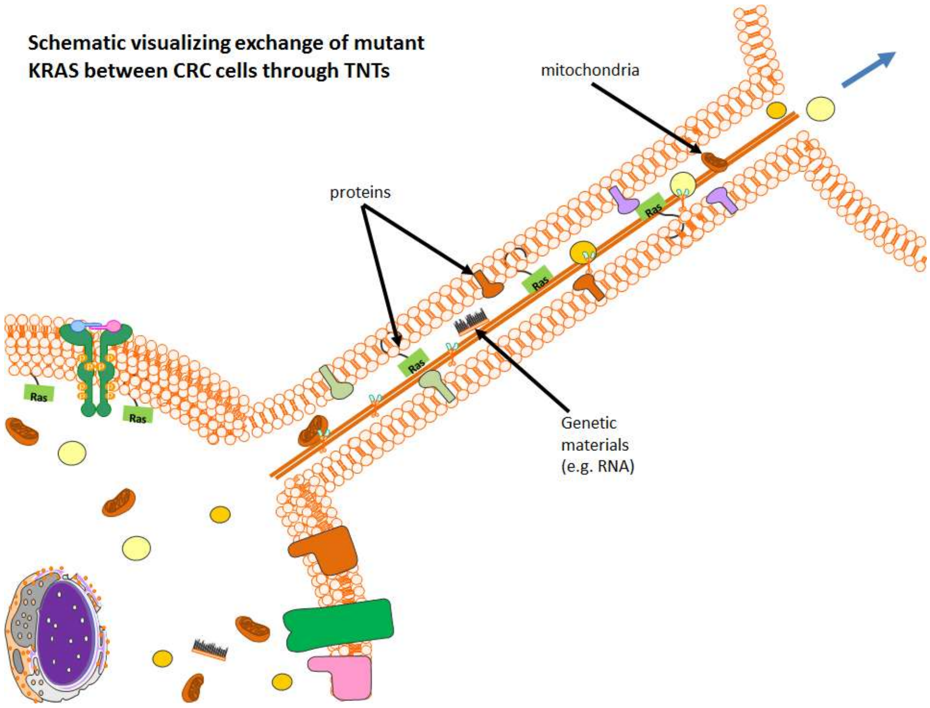

Figure S7. Schematic visualizing the exchange of mutant KRAS between CRC cells through TNTs.

Video S1: Time-lapse microscopy movie of FRAP-based mutant KRAS transport analyzed within a TNT.

Video S2: Time-lapse microscopy movie of FRAP-based mutant KRAS transfer analyzed at the LOVO TNT and HCT-8 cell membrane interface.

Video S3: Time-lapse microscopy movie of JF646 dyed HALO tagged KRAS moving within cellular protrusions.

Video S4: Time-lapse microscopy movie of HCT-8 cell sharing GFP mutant KRAS protein via a TNT.
